# Missense mutation of *SERPINC1* (p.Ser426Leu) in a young patient presenting as refractory and recurrent venous thromboembolism: A case report

**DOI:** 10.3389/fcvm.2022.903785

**Published:** 2022-08-24

**Authors:** Haixu Yu, Xiaoyan Gai, Jianli Wang, Jinman Zhuang, Wanwan Guo, Rui Qiao, Hong Zhu, Yongchang Sun

**Affiliations:** ^1^Department of Pulmonary and Critical Care Medicine, Peking University Third Hospital, Beijing, China; ^2^National Health Commission (NHC) Key Laboratory of Cardiovascular Molecular Biology and Regulatory Peptides, Key Laboratory of Molecular Cardiovascular Science, Ministry of Education, Beijing Key Laboratory of Cardiovascular Receptors Research, Department of Cardiology and Institute of Vascular Medicine, Peking University Third Hospital, Beijing, China; ^3^Department of Interventional Vascular Surgery, Peking University Third Hospital, Beijing, China; ^4^Department of Laboratory Medicine, Peking University Third Hospital, Beijing, China

**Keywords:** antithrombin deficiency, *SERPINC1*, venous thromboembolism, missense mutation, case report, refractory and recurrent VTE

## Abstract

Genetic and acquired risk factors are extremely important mechanisms in the development of venous thromboembolism (VTE). Inherited antithrombin (AT) deficiency due to mutations in the *SERPINC1* gene is a well-known risk factor for genetic thrombophilia. In this case, we reported a 28-year young abroad student who presented with refractory and recurrent VTE in-hospital. This patient presented with a 2-month history of right lower limb pain and 1 week of fever. The ultrasound showed deep venous thrombosis in the right common and superficial femoral veins. The CTPA confirmed acute pulmonary embolism with multiple filling defects in both pulmonary arteries. He was diagnosed with “pulmonary embolism, pneumonia, lower extremity venous thrombosis”. The level of serum antithrombin was normal, yet gene sequencing revealed a heterozygous missense mutation of *SERPINC1*, c.1277C>T (p.Ser426Leu). The patient underwent anticoagulant therapy of heparin and inferior vena cava filter implantation. The patient had undergone recurrent VTE despite adequate anticoagulation with heparin during the first 2 weeks. The swelling, pain, and thrombosis of lower extremity veins got resolved from warfarin and rivaroxaban. Inherited antithrombin deficiency due to mutations in the *SERPINC1* gene is the genetic basis of this patient, and warfarin/rivaroxaban, other than heparin, is beneficial.

## Introduction

Venous thromboembolism (VTE) is an acknowledged multifactorial disease that contributes significant burden on health and survival, usually resulting from inherited and acquired risk factors (1). Inherited risk factors can predispose patients to thrombophilia including deficiencies of antithrombin (AT), protein C, and protein S, along with factor V Leiden mutation and prothrombin G20210A mutation (2). The common acquired factors (e.g., age, obesity, sedentariness, immobility, infection, inflammation, surgery, trauma, and use of oral contraceptive) may trigger the occurrence of VTE on an inherited thrombotic terrain. As a member of the serine proteinase inhibitor (SERPIN) superfamily, AT acts as the pivotal plasma inhibitor of thrombin and other coagulation proteases. Although inherited AT deficiency is a rare (1/2,000–1/3,000 individuals) autosomal dominant disease that is caused by mutations in the encoding gene (*SERPINC1*) at chromosome band 1q23-25, the prevalence of AT deficiency unexpectedly accounts for 1–5% in patients with VTE (3). Here we present a young man in-hospital presenting with refractory and recurrent venous thromboembolism with heparin anticoagulation. Gene sequencing revealed a missense mutation of *SERPINC1* (p.Ser426Leu).

## Case presentation

**Figure 1:**
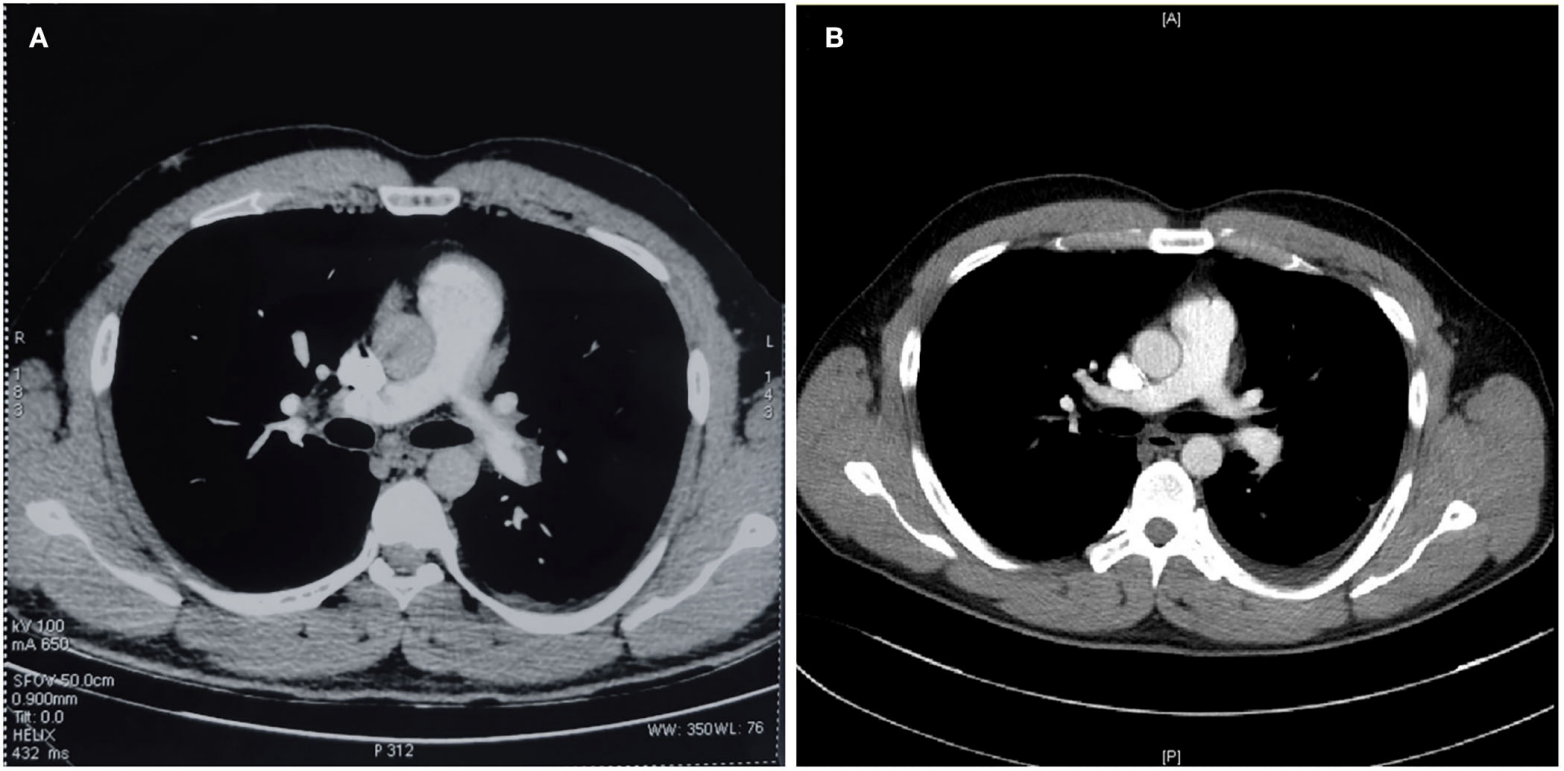
CTPA demonstrated confirmed pulmonary embolism [**(A)** CTPA scan before admission] and comparison of therapeutic effects [**(B)** CTPA scan before discharge].

As an international doctoral student from Uzbekistan, this previously healthy 28-year-old male was admitted to our hospital with right lower extremity pain and fever. Two months ago, the patient developed pain and swelling in the right lower extremity after sitting for a long time. Ten days ago, he was admitted to one local hospital because of a fever with cough. The blood test showed elevated white blood cells, D-dimer (9.59 μg/ml,↑) and mycoplasma pneumonia IgM (+). The ultrasound showed deep venous thrombosis in the right common and superficial femoral veins. The CTPA confirmed acute pulmonary embolism with multiple filling defects in both pulmonary arteries (Figure 1A). Then he was diagnosed with VTE and pneumonia with the treatment of low molecular weight heparin (LMWH), inferior vena cava filter, and antibiotics (moxifloxacin), and admitted to the respiratory intensive care unit (RICU) in our hospital (2021-03-14). All the families had no VTE phenotypes and no family history of venous thrombosis. And this obese young Caucasian patient (BMI = 30.1) had a sedentary lifestyle because of struggling with research work (10 h/day).

### Laboratory instruments

Detection for coagulation test, anti-Xa, and AT activity were measured using an automatic coagulation instrument (ACL-TOP^®^, Instrumentation Laboratory, Spain). The automated chromogenic assay for the quantitative determination of AT in human citrated plasma was collected by venipuncture of an antecubital vein into vacuum tubes (Becton Dickinson Medical Devices Co Ltd., Franklin Lakes, New Jersey) containing 1:9 volume of 0.109 mol/L sodium citrate. Cytokines were detected by flow cytometry.

### Thrombophilia screening

AT-III, protease C, protease S, and anti-cardiolipin antibody combinations were not significantly abnormal; anti-phosphatidylserine/prothrombin (aPS/PT) antibodies were normal; anti-nuclear antibody and antinuclear antibody profiles, and anti-neutrophil cytoplasmic antibody were negative.

### Lupus anticoagulant test

SCT screening test 28s↓, SCT validation test 35s, standard SCT ratio 0.69, dRVVT screening test 39s, dRVVT validation test 30s, standard dRVVT ratio 1.24↑.

Anti-Xa and AT activity tests are shown in Table 1.

**Table 1:** Anti-Xa and AT activity test results.

	**Anti-Xa (IU/ml)**	**APTT (s)**	**AT (%)**
0.5 h before heparin withdrawal	0.68	81	77
1 h before heparin withdrawal	0.45	56.9	79
0.5 h after heparin administration	0.25	39.3	83

Anti-Xa and AT activity were tested during continuously unfractionated heparin pumping (2,300–3,000 U/h = 27–35 U/kg^*^h).

### Lipids

Total cholesterol 5.46 mmol/L↑, triglycerides 1.82 mmol/L↑, HDL cholesterol 0.61 mmol/L↓, LDL cholesterol 4.2 mmol/L↑, ApoA1 0.85 g/L↓, ApoB 1.15 g/L, lipoprotein (a) 36 mg/L; ultrasensitive C-reactive protein 32.9 mg/L↑.

### Coagulation test (admission)

Fibrin degradation products (FDP) 80.7 μg/ml↑, D-dimer 9.59 μg/ml↑; homocysteine 17.1 umol/l↑.

### Infective index

Mycoplasma pneumoniae antibody quantification 1:40 positive; tumor biomarkers, free PSA, total PSA, AFP, CA724, CA199, G test, GM test, PCT were all negative; glycated hemoglobin, cTnT, BNP, liver and kidney function, electrolytes, thyroid function, stool routine, ECG, abdominal ultrasound, and echocardiogram did not show any significant abnormalities.

### Genetic analysis

The Next-generation sequencing (NGS)-based custom gene panel of 126 genes associated with inherited bleeding and thrombotic disorders was performed on MGISEQ-2000 instrument using 2 × 151 bp paired-end mode. The capture design includes all coding sequences (CDS) and 5'and 3' UTRs in Ensembl and RefSeq. In total, 367,167 bp were targeted. DNA was extracted from blood and its quality and concentration were assessed by Qubit3.0 measurement. The libraries were sequenced by the Department of Hematology, Peking Union Medical College Hospital. The mean depth of each sample was 1,000 ×. Single nucleotide variants (SNVs) and insertions/deletions (indels) were called using GATK4 HaplotypeCaller.

**Figure 2:**
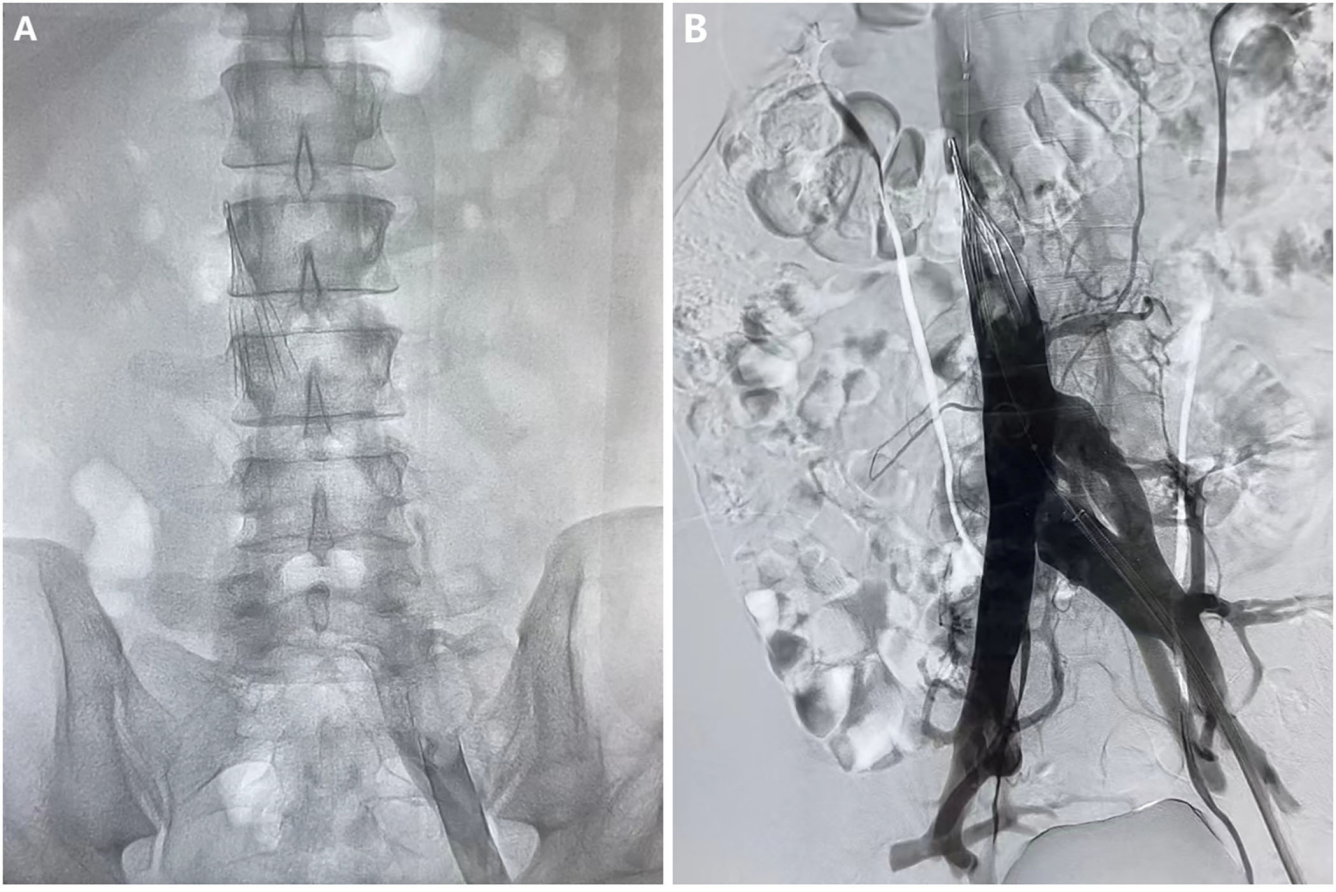
Angiography of vena cava before **(A)** and after **(B)** the thrombectomy and thrombolytic therapy.

**Figure 3:**
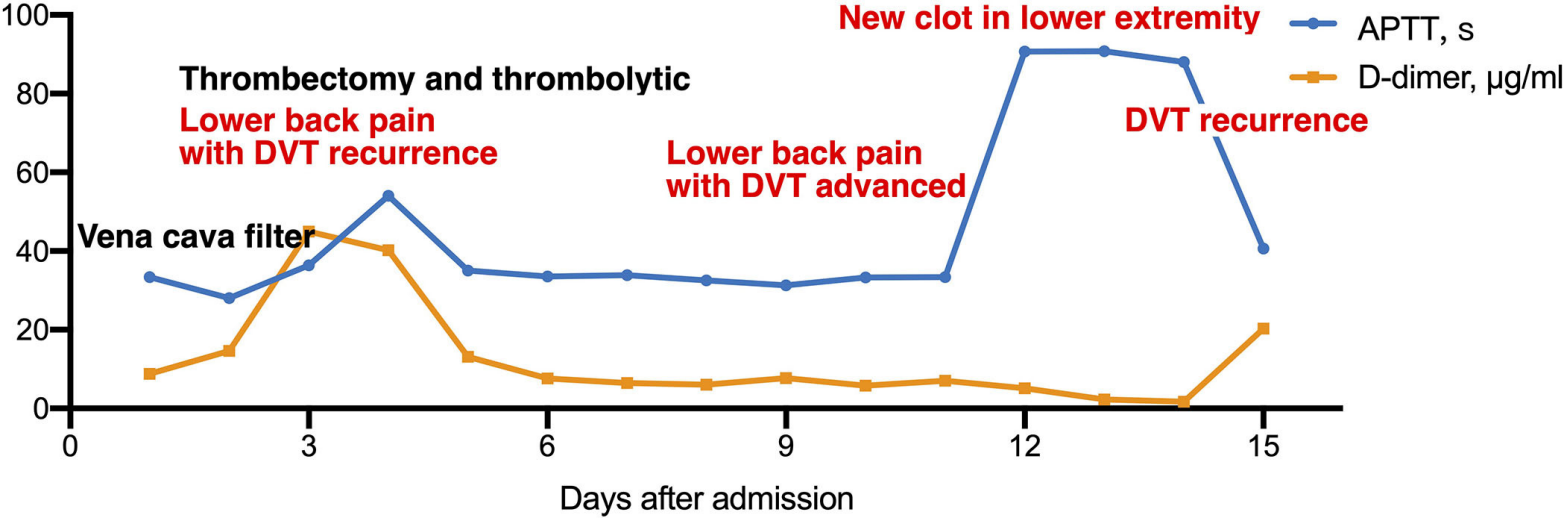
The timeline during the treatment in hospital and coagulation tests.

After admission to RICU, the patient was treated with LMWH (enoxaparin sodium, CLEXANE) for 8,000 units per 12 h and moxifloxacin 400 mg per day. As the condition gradually stabilized, he was transferred to the respiratory department 2 days later. On 2021-03-16 afternoon, the patient developed lower back pain, elevated D-dimer, worsening venous thrombosis in both lower extremities, and obstruction in the vena cava filter. On 2021-03-17, he underwent vena cava angiography, thrombectomy, and thrombolytic therapy in the interventional vascular department (Figures 1B, 2A, 2B). In order to avoid recurrence of thrombosis, he was treated with a higher dose of LWMH for 9,000 units per 12 h for anticoagulation (1.05 mg/kg). A Thrombophilia gene mutation test was taken. On 2021-03-23, the patient complained of lower back pain with D-dimer elevation. The ultrasound showed bilaterally external iliac vein, common femoral and right superficial femoral, popliteal, and intermuscular vein thrombosis, which was progressed than before. Then the patient was treated for warfarin 3 mg per day initially. The INR of the coagulation test didn't reach the target (2.5–3.0). The warfarin pharmacogenomic polymorphism test showed the VKORC1 gene (−1,639 G>A) alternation. Thus, a higher dose of warfarin (6 mg qd) was treated. On 2021-03-25, the patient complained of severe pain in his left lower extremity, and ultrasound suggested bilateral thrombosis of the external iliac vein, common femoral vein, superficial femoral vein, popliteal, and intermuscular vein. Then unfractionated heparin (UFH) was continuously pumped to maintain APTT for 60–90 s (2,300–3,000 U/h = 27–35 U/kg^*^h), while warfarin 6 mg per day was administered. Anti-Xa assay showed normal activity of AT antigen (Table 1). On 2021-03-28, the patient complained of advanced pain in the left lower extremity again, and he had recurrent thrombosis in both lower extremity veins on ultrasound. On 03–29, his INR reached 4.0, and the dosage of warfarin was adjusted to 6 to 4.5 mg qod. There were significantly elevated IL-1β (24.87 pg/ml, normal range ≤ 12.4 pg/ml), IL-6 (42.6 pg/ml, normal range ≤ 5.4 pg/ml) and IL-8 (1,080.03 pg/ml, normal range ≤ 20.6 pg/ml) in serum. Considering a high-level of CRP and LDH reflected intense inflammatory activation, low-dose glucocorticoid (12 mg/d) was treated for 3 days. The CTPA was reviewed again and showed well therapeutic effects (Figure 1B). Due to inadequate medical insurance, he returned to Uzbekistan for treatment. The timeline during the treatment in the hospital was reorganized in Figure 3.

### Follow-up

After he arrived in Uzbekistan, the physician supplied the pulse steroid therapy for him (500 mg/d for 2 days) and continued warfarin anticoagulation. The lower extremity swelling and pain were relieved than before. During short-term warfarin anticoagulation, then the strategy was adjusted to long-term rivaroxaban of 20 mg per day. The filter was not removed for 6-month. The thrombosis event did not happen anymore. NGS revealed a heterozygous cytosine to thymine substitution at nucleotide position 1277 in exon 7 of the *SERPINC1* gene, replacing serine with leucine on codon 426 (c.1277C>T; p.Ser426Leu). This mutation is included in the *SERPINC1* mutation database of the NCBI (rs121909550) and HGMD (CM890019).

## Discussion and conclusion

In this case, we reported a young man presenting with refractory and recurrent venous thromboembolism with pseudomorph of normal AT. Genetic detection showed missense mutation of *SERPINC1* (c.1277C>T) was identified in exon seven and resulted in the substitution of Ser426 with Leu. He underwent recurrent VTE with Heparin anticoagulation. After warfarin anticoagulation and rivaroxaban treatment, subsequently, no adverse events occurred during the 6-month follow-up.

VTE is well-known as a multicausal disease influenced, the multiply inherited and acquired factors participated important role in the occurrence and prognosis of this disease. In terms of involvement in pathophysiological processes, genetic factors can be classified into two main categories (1) functional inhibition of endogenous anticoagulants (deficiency/dysfunction of protein C, protein S, and antithrombin); (2) functional enhancement of procoagulant factors (PT20210A and factor V Leiden). Although the majority of patients with VTE should not be regularly tested, patients in conjunction with weak predisposing factors at a young age, family history, and recurrent VTE events have a high likelihood of inherited thrombophilia (4). Besides various acquired factors such as obesity, sedation, immobility, infection, inflammation, hyperhomocysteinemia, and hypercholesterolemia, this young patient possessed a genetic mutation background in *SERPINC1*, which is a causative effect of VTE. The previous meta-analysis showed ATD increased 16-fold risk for first VTE and 4-fold risk for recurrence, approximately (5). According to the decreased level of circulating protein, inherited AT deficiency (ATD) is characterized by Type I (quantitative) and Type II (qualitative) which the latter composed of three different subgroups due to mutations site (reactive site, heparin-binding site, and pleiotropic effects) (6, 7). Type I ATD is manifested by a reduction of both plasmas AT activity and antigen levels. The pathogenesis for Type I is the insertion or deletion of bases in the *SERPINCl* gene, which affects the synthesis of mRNA and then prevents hepatic cells from the synthesis of antithrombin (8). Type II ATD is manifested by normal levels of AT but reduced AT activity. Based on functional defects, Type II ATD can be further distinguished into three subtypes: type II RS (reactive site), when the mutation has impaired the reactive center loop. The ability to inactivate thrombin and factor Xa is greatly reduced; type II HBS (heparin-binding site), where the mutation site is located in the heparin-binding domain and affects the interaction between heparin and antithrombin; type II PE (pleiotropic effect), where the mutation affects both the reactivity and heparin affinity (9). On the basis of phenotype (youth and recurrent VTE), laboratory test (Anti-Xa level and AT activity, Table 1), and gene sequencing result (missense mutation of *SERPINC1*, p.Ser426Leu), the subgroup of ATD might be Type II (reactive site) which resulted in the ability to inactivate thrombin and Xa significantly decreased (6). Thus, the anticoagulant effect for heparin and LMWH was unsatisfactory, during warfarin and rivaroxaban, moderate thrombosis event. Patients with normal AT levels are easily neglected in the clinic. It should be alerted to the probability of AT mutations in recurrent VTE patients, and prompt genetic testing should be performed even if AT is normal.

Cases with transient antithrombin deficiency have very recently been described (10, 11). Inflammatory processes are always orchestrated by pleiotropic and multifunctional cytokines and chemokines which have pro-thrombotic effects in thrombosis (12). Previous studies have demonstrated that the coagulation activation markers IL-1β, IL-6, and IL-8 were associated with VTE (13). The pathophysiology mechanisms were correlated with platelet activation, neutrophil activation, and hypoxia-related hypercoagulation. Although glucocorticoids are widely used agents for anti-inflammatory and immunosuppressive therapy, several side effects, including increased risk of VTE and cardiovascular complications (myocardial infarction and stroke) still a matter of controversy. For this patient, the anti-inflammatory treatment appears to be the brake of the coagulation cascade, so that anticoagulant prevented continuous VTE recurrence. However, relatively therapeutic evidence is still insufficient.

## Conclusion

In clinical practice, antithrombin deficiency faces the dilemma of underestimation, including the transient antithrombin deficiency. Traditional diagnostic approaches born with false negatives in thrombophilia. Timely genetic testing helps to identify the pathogenic mutations and facilitates the selection of anticoagulant drugs, which is important for treatment and prognosis.

## Data availability statement

The original contributions presented in the study are included in the article/supplementary material, further inquiries can be directed to the corresponding author.

## Ethics statement

Ethical review and approval was not required for the study on human participants in accordance with the local legislation and institutional requirements. The patients/participants provided their written informed consent to participate in this study. Written informed consent was obtained from the individual(s) for the publication of any potentially identifiable images or data included in this article.

## Author contributions

WG and RQ performed the AT examination. JZ did the operation and provided the photos. HY and XG were major contributors in writing the manuscript. All authors analyzed and interpreted the patient data. All authors read and approved the final manuscript.

## Funding

This work was supported by the Capital's Funds for Health Improvement and Research [2022-2G-40910, and National Natural Science Foundation of China Youth Fund Project (No. 81400038)].

## Conflict of interest

The authors declare that the research was conducted in the absence of any commercial or financial relationships that could be construed as a potential conflict of interest.

## Publisher's note

All claims expressed in this article are solely those of the authors and do not necessarily represent those of their affiliated organizations, or those of the publisher, the editors and the reviewers. Any product that may be evaluated in this article, or claim that may be made by its manufacturer, is not guaranteed or endorsed by the publisher.
